# The Prognostic Value of Lymph Node Cross-Sectional Cancer Area in Node-Positive Breast Cancer: A Comparison with N Stage and Lymph Node Ratio

**DOI:** 10.1155/2012/161964

**Published:** 2012-10-04

**Authors:** Yanxia Li, Earle Holmes, Karan Shah, Kevin Albuquerque, Anna Szpaderska, Çağatay Erşahin

**Affiliations:** ^1^Department of Pathology, Loyola University Medical Center, 2160 S First Avenue, Maywood, IL 60153, USA; ^2^Department of Surgery, Loyola University Medical Center, 2160 S First Avenue, Maywood, IL 60153, USA

## Abstract

The number of positive axillary lymph nodes (LNs) is the only node-related factor for prognostic evaluation of breast cancer recognized by AJCC (TNM staging). However, N staging may not completely reflect LN tumor involvement due to the erroneous count of LNs in the presence of matted LNs and different tumor volume in LNs. Additionally, the positive/total LN ratio (LNR) has been shown to outperform N staging in survival prediction. In our study, to better quantify the tumor involvement of axillary LNs, we measured the cross-sectional cancer area (CSCA) of the positive LNs in 292 breast cancer patients diagnosed between 1998 and 2000 in our institution and compared its prognostic value to that of number of positive LNs (metLN)/N stage and LNR. Statistical analyses of these three LN-related factors were performed by Kaplan-Meier method and multivariate Cox's regression model. Patients were divided into three groups based on the different LN CSCA (<50, 50–500, and >500 mm^2^), or LNR (<0.1, 0.1–0.65, and >0.65), or N stage (N1–N3). Multivariate analysis demonstrated LNR was the most significant LN-related survival predictor with hazard ratio (HR) 25.0 (*P* = 0.001), compared to the metLN (HR 0.09, *P* = 0.052) and CSCA (HR 2.24, *P* = 0.323).

## 1. Introduction

Breast cancer was the most common malignancy in women in North America in 2010 [1]. The involvement of axillary LNs by cancer is one of the most important factors for cancer staging, treatment, and prognosis [2–5]. The surgical excision of the primary cancer and the axillary LN dissection has been considered as part of the standard management of invasive breast cancer [6–8]. Counting the number of positive axillary LNs was used for TNM staging [9], and it is the only node-related factor for the evaluation of breast cancer recognized by American Joint Committee on Cancer (AJCC) [10]. In general, evaluating 10 or more LNs is ideal for accurate assessment and the staging of breast cancer [6, 7]. Besides LN staging, other equally important prognostic factors associated with breast cancer are tumor size, histological grade, and hormone receptor status [11]. According to AJCC, based on the number of positive LNs (metLN), patients are divided into three N stages: N1 (1–3 positive LNs), N2 (4–9 positive LNs), and N3 (>9 positive LNs). There are some considerations of minimal tumor involvement such as isolated tumor cells (<0.2 mm or <200 tumor cells) and micrometastasis (>0.2 mm and/or >200 tumor cells and <2 mm) in N0 and N1, respectively [9]. However, the quantitative criteria have not been considered in the AJCC staging system in positive LNs with cancer involvement greater than 2 mm. For example, every involved LN is counted as positive without regard to the volume of tumor which ranges from a small microscopic focus to a near total replacement of the entire LN. In addition, there is no good way of handling a large matted LN in the current pathologic TMN staging system, even though clinical stage N2 is applied with the presence of matted LNs [10]. In these situations, the metLN may not completely reflect the degree of tumor involvement in the LNs. To address this problem, we quantified the metastatic tumor volume by measuring cross-sectional cancer areas (CSCAs) in the positive axillary LNs using computer imaging system.

The positive LN ratio (LNR, defined as the ratio of the metLN to the total number of LNs examined) or the percentage of positive axillary LN was recently reported to be a strong predictor of breast cancer survival by several studies [12–21]. Multivariate analysis in these studies showed that LNR typically outperformed N stage in predicting survival of breast cancer patients. Our study evaluated three node-related factors: metLN/N stage, LN CSCA, and LNR, and their association with prognosis. Our goal was to retrospectively compare these different methods and to identify the most significant LN-related predictor of breast cancer survival. We also evaluated other risk factors including age, tumor size, T stage, histological grade, hormonal status, and extracapsular extension (ECE) of axillary LNs using univariate and multivariate analysis.

## 2. Materials and Methods 

**Figure 1:**
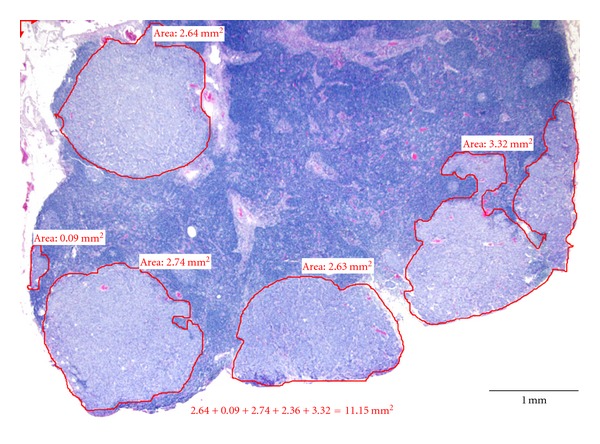
The screenshot of measurement of LN CSCA using Software Imaging System Olympus MicroSuite 5. Multiple foci of metastatic cancer were outlined. The size of individual cancer areas and the sum of all areas were calculated by the imaging software.

**Figure 2:**
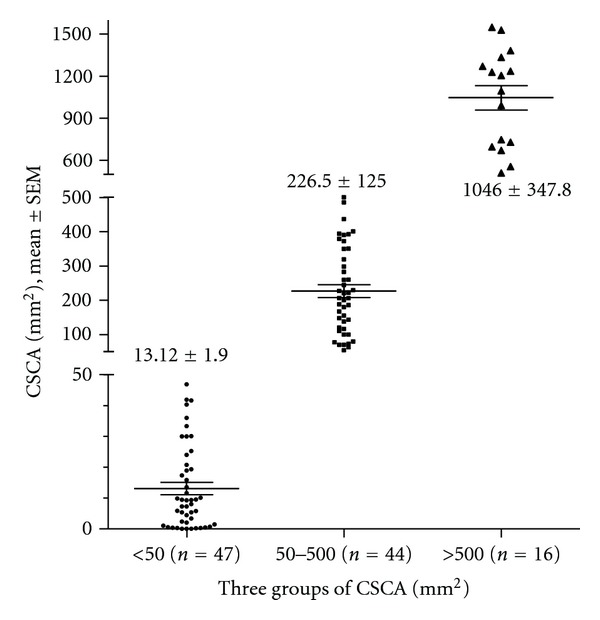
Scatter plot with mean ± SEM to show the details of LN CSCA distribution in the three groups, <50, 50–500, >500 mm^2^.

**Figure 3:**
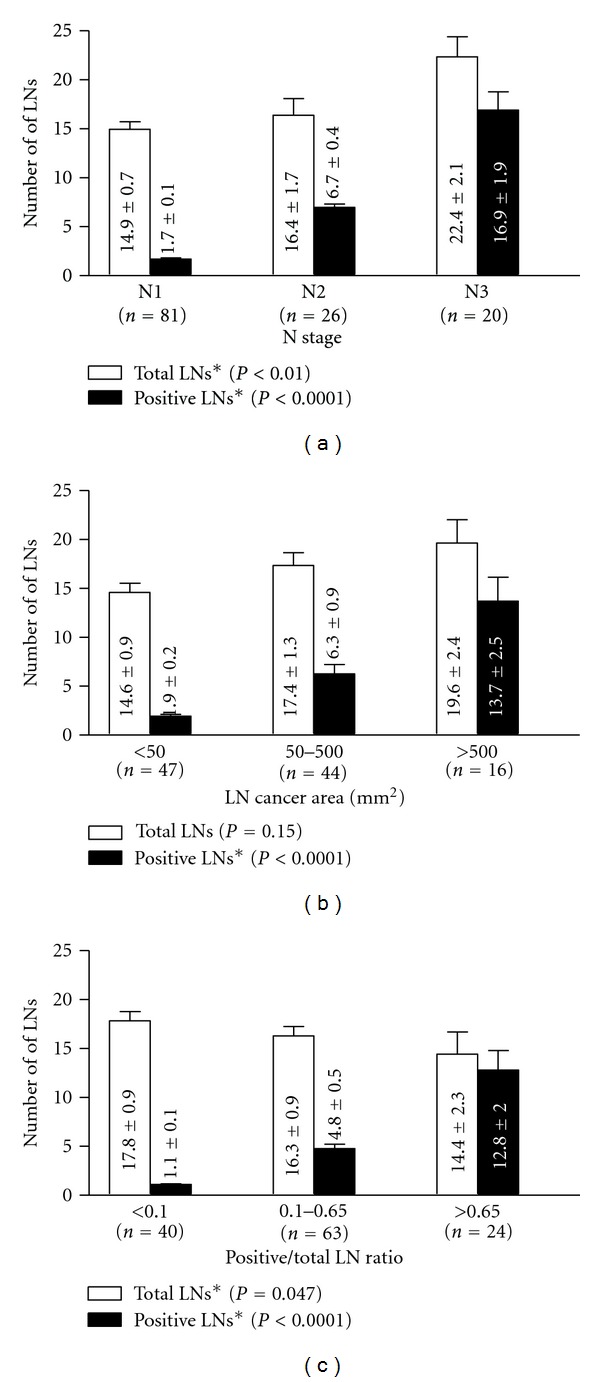
Comparison of the number of positive LNs (metLN) and total LNs examined in the three methods N stage, LN CSCA, and LNR. ∗Statistically different with *P* < 0.05.

The surgical reports and the medical records of 292 breast cancer patients diagnosed between 1998 and 2000 in our institution were retrospectively analyzed. The time frame of 1998–2000 is selected in that it allows at least a 10-year followup of the survival data. Information gathered for each patient includes age, tumor characteristics such as histologic grade, tumor size, T stage, metLN, N stage, total number of LNs examined, estrogen (ER) and progesterone receptor (PR) expression of tumors by immunohistochemical stains, and ECE of positive LNs. All the tumors were graded according to the Nottingham combined histologic grade. All the LNs are either bisected or serially sectioned into 2 mm thickness and submitted for histologic examination. ECE is defined by the clear penetration of cancer cells through the capsule of the LNs. The extent of metastatic cancer involving LNs was quantified in mm^2^ by measuring the area of cancer in these LNs (using Software Imaging System Olympus, MicroSuite 5, Pathology Edition). A screenshot of the cancer area measurement on a cross-section of an LN using the software is demonstrated in Figure 1. The contour of the cancer areas was outlined by a “pencil” tool in the program. If there were multiple foci of the metastatic tumor in a cross-section, the program calculated the individual contoured areas and then automatically summed all of them to calculate the total area. For positive LNs that were sectioned into multiple pieces, the measurement was selectively performed in one representative cross-section of the LN with the largest cancer area. If there are multiple positive LNs identified, the sum of CSCA in all positive LNs is obtained. Among 127 node-positive patients, 107 patients had available histologic slides for assessing the LN CSCA. These patients were divided into 3 groups based on the measured LN cancer areas: (1) <50 mm^2^, (2) 50–500 mm^2^, and (3) >500 mm^2^. The distribution of CSCA is illustrated in a scatter plot with mean ± SEM shown (Figure 2). The LNR was expressed as the ratio of metLN to total LNs examined. 127 node-positive patients were divided into three groups based on LNR: (1) 0.1, (2) 0.1–0.65, and (3) >0.65. metLN and total numbers of LNs are compared among different groups of N stage, LN cancer area, and LNR (Figure 3). 

## 3. Statistical Analysis

Statistical analysis of LN numbers (Figure 3) was performed by a one-way ANOVA test (Kruskal-Wallis nonparametric test, and Dunn's tested was used for pairwise comparison within the group) using GraphPad Prism 5.04 (GraphPad Software, Inc.). The results are expressed as mean ± SEM. Data from 107 patients with completely paired metLN/N stage, CSCA, and LNR were used for the univariate survival analyses by Kaplan-Meier method and multivariate Cox's regression model [18]. Overall survival was analyzed with fatality from any cause as the end point. Differences between groups were evaluated using the log-rank test. In the multivariate analyses performed using Cox's regression model, some covariates were treated as continuous variables including metLN, LN CSCA, LNR, age, and tumor size. The others were modeled as binary variables including histologic grade (grade 1 and 2 versus grade 3), T stage (T1 versus T2, T3 and T4), ECE (yes versus no), and ER status (yes versus no). MetLN, CSCA, and LNR were standardized within the range of 0 to 1. Multivariate analysis was performed using SPSS 16.0.1. statistical software (SPSS, Chicago, IL). 

## 4. Results

292 patients with breast cancer diagnosed from 1998 to 2000 in our institution were included in this study. The patient characteristics are summarized in Table 1. The mean age of the patients was 59.0 ± 0.82 (mean ± SEM) years ranging from 26 to 93 years. The median followup was 8.33 years, with a maximum followup of 10.92 years. 74 patients (25.3%) died during the period of study. Information on N stage, T stage, histologic grade, ECE, tumor hormonal status, LNR, and LN cancer area is also provided. The number of total LNs examined and metLN are compared in Figure 3 according to N stage, LN CSCA, and LNR, with the mean ± SEM listed. The metLN increased in all three categories: from N1 to N3 in N stage, from low to high LN CSCA and LNR (*P* < 0.0001). Interestingly, the number of total LNs examined was increased within the groups of N stage (*P* < 0.01), did not show statistical difference in LN CSCA (*P* = 0.15), and however decreased with increasing LNR (*P* < 0.05). 

The actual 5-year survival rate was 82%. A univariate analysis for 5-year survival rate is shown in Table 2. Histologic grade (*P* = 0.001), N stage (*P* < 0.0001), T stage (*P* < 0.0001), ER expression (*P* < 0.0001), ECE (*P* < 0.0001), LN CSCA (*P* < 0.0001), and LNR (*P* < 0.0001) were all found to be predictive for overall survival. Age (50 years was treated as cutoff) did not affect overall survival (*P* = 0.6). Multivariate analysis (Table 3) revealed LNR was the most significant prognostic predictor of breast cancer mortality with a hazard ratio of 25.0 when LNR was scaled from 0 to 1. In addition, histologic grade and ER expression were found to be independent and significant prognostic factors of breast cancer survival. In different models, we tested the prognostic significance of individual LN-related factor by multivariate analysis. When CSCA, N stage (categorical data, N1 versus N2 + N3), or LNR was present as the only LN covariate along with other significant variants (ER, age, T stage, tumor size, ECE, and tumor grade), each of them was demonstrated as an independent predictor (*P* = 0.037,  *P* = 0.006, and *P* < 0.001, resp.). The result failed to show the metLN (continuous data) as an independent predictor when present alone (*P* = 0.099). When both CSCA and metLN (or N stage) coexisted in the model, neither showed prognostic significance (*P* = 0.097 and *P* = 0.868, resp.). When all three LN covariates were entered in the model, only LNR remained as a significant predictor for survival (Table 3). 

**Figure 4:**
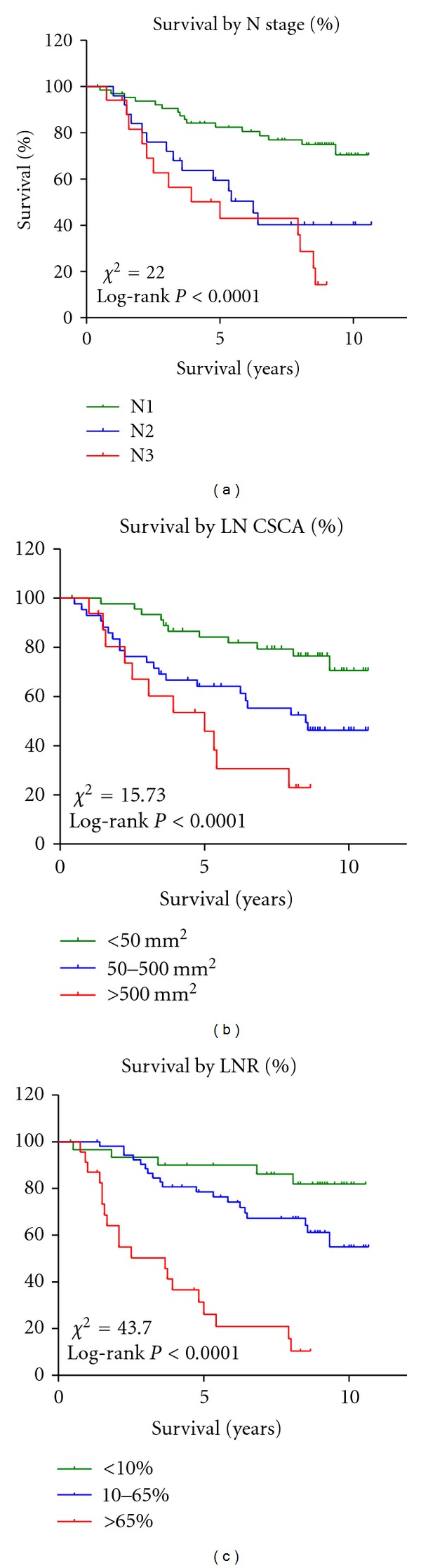
The Kaplan-Meier survival analysis of node-positive patients (*n* = 107) according to N stage, LN CSCA, and LNR.

The univariate Kaplan-Meier analysis of 107 node-positive patients grouped according to the three LN-related parameters (N stage: N1–3, LN CSCA: <50, 50–500, and >500 mm^2^, and LNR: <0.1, 0.1–0.65, and >0.65) is shown in Figure 4. Survival curves of groups N2 and N3 overlapped and crossed between 5 and 10 year. In contrast, survival curves based on the LN CSCA and the LNR analysis demonstrated distinct separation of these groups.

## 5. Discussion

The metLN, tumor size, histologic grade, and hormonal receptor expression status are reported to be the main prognostic factors associated with breast cancer [11]. As the metLN is the only LN-related prognostic factor recognized by AJCC [9], it is reasonable to question whether it is the most accurate way to represent the status of metastatic cancer in the LNs.

The dimension of tumor involvement in positive LNs has been distinguished in stages N0 and N1 as isolated tumor cells, tumor clusters, and micrometastasis by AJCC LN staging system [9]. Survival analyses of the breast cancer patients with isolated tumor cells and micrometastasis in the sentinel lymph nodes showed controversial results [22–27]. There is no further distinction of tumor dimension above 2 mm in the LNs, whereas only the metLN is taken into account. In other words, an LN with just a small focus of subcapsular tumor invasion and an LN with a near total replacement by the tumor are both counted as one positive LN. In addition, there is the dilemma that counting matted LNs may render a source of variability in determining the pathologic N stage [10]. Although the largest dimension of the positive LN was recorded in our staging summary and can be used to reflect the extent of tumor involvement in cases of the matted LNs, this measurement has never been evaluated as a variable in the TNM staging system. To address these issues, we initially hypothesized that measuring the CSCA of the LNs instead of counting the metLN would give a more accurate picture of the metastatic cancer volume in the axillary LNs. The present state of computer technology allows us to measure the CSCA accurately and objectively. 

Previously, LN CSCA has been studied in a variety of cancers. For example, in melanomas, LN CSCA has been found to correlate with the Breslow thickness and the likelihood of further nodal involvement in completion of LN dissection [28, 29]. Percentage of CSCA in sentinel LNs in breast cancer has also been evaluated and reported to be the most important predictor of frequency of additional positive nonsentinel LNs in multivariate analysis [30]. However, there is prognostic analysis based on CSCA in this study of breast cancer. Early study on esophageal cancer by Komori et al. has reported the size of the largest cancer nest in the LNs is one of the most important prognostic factors [31]. Recently, growing evidence and a large body of literatures summarized by Petrelli et al. [32] on esophageal and other gastrointestinal cancer have demonstrated that LNR is an independent powerful predictor of estimated survival.

In our study, 292 breast cancer patients diagnosed from 1998 to 2000 were included for the evaluation of prognostic factors of breast cancer, allowing a sufficient time frame for follow-up study. Univariate Kaplan-Meier analysis demonstrated LN CSCA was a better prognostic indicator than N stage where N2 and N3 overlapped and crossed (Figure 4). This indicates that patients with greater than 4 positive LNs (N2 and N3) showed similar survival without significant difference. There are other studies that support this observation [5, 10]. We also evaluated different cutoff points of LN CSCA, but none of them appeared to be able to categorize patient's survival very distinctively. Multivariate Cox's regression model demonstrates that CSCA (but not metLN) is an independent prognostic indicator when present alone without other LN factors. However, when both CSCA and metLN are present in the model, neither turns out to be an independent predictor. Furthermore, we thoroughly compared the LN CSCA method with N staging method in terms of cancer definition including metLN, histologic grade, T stage, and ECE (data not shown). These two methods were similar in characterizing the breast cancer, and they are both dependent on the metLN. Our results indicate that the quantitative LN CSCA can serve as an alternative to the AJCC's current LN staging system. 

LNR in breast cancer has been studied in recent years and proposed to be a promising prognostic factor that outperforms the currently used LN staging scheme. Vinh-Hung et al. reviewed and summarized the latest study on the prognostic value of LNR in 2009 [18]. The variation of the LN dissection and the inconsistency of axillary LN evaluation are caused by a biological variation in patients as well as different techniques across institutions [33]. In addition, some patients who were staged with only one sentinel LN underwent neoadjuvant chemotherapy without axillary dissection [2, 7, 34]. Thus, the N stage represented by the absolute metLN may not be a universal indicator of prognosis in breast cancer. For example, the difference between 3/3 (3 positive out of 3 total LNs examined) and 3/20 LNs (3 positive out of 20 total LNs examined) could be due to technical variations from different surgical approaches. Given the small denominator, it is unclear whether it is possible to harvest more LNs in the first scenario (3/3), and how many LNs would turn out to be positive had they been harvested. Both cases are categorized as N1 tumor based on the metLN. In contrast, when the total number of LNs is considered in the calculation of LNR, the cases have LNRs 1.0 (3/3) and 0.15 (3/20), respectively, and will likely fall into different LNR categories depending on the cutoff points applied.

We added LNR into our group of LN-related factors for the prognostic comparison. We adapted the analytical method used by Vinh-Hung et al. for our data analysis [18]. Log-rank test demonstrated significantly different survival among three LNR groups (<0.1, 0.1–0.65, >0.65) (Figure 4). In our multivariate analysis, LNR was the only independent significant prognostic predictor for cancer mortality among all three LN-related factors (Table 3). 

We further evaluated the lack of agreement among the three different categories in survival prediction by comparing metLN and total LN examined (Figure 3). Interestingly, our observation revealed that the metLN in all methods increased within the groups, while the total number of LNs examined increased from N1 to N3, stayed the same in the LN CSCA groups, and decreased from low to high LNR groups. Thus, taking the ratio of positive over total LNs is a powerful method with the acknowledgment of total LNs as an “internal control,” balancing off the unevenness from counting only the absolute metLNs. 

A comprehensive assessment of all aspects of breast cancer diagnosis is beyond the scope of this paper. The current study takes the advantage of single patient population treated by one surgical team using consistent surgical procedures within a 3-year time period. This allows a retrospective comparison focusing on the different LN-related factors with the minimal variability among other parameters. The automated programs made the cancer area measurement simple and quick. To the best of our knowledge, it is the first study that compared three LN-related methods (N stage, LNR, and LN CSCA) on the same population of breast cancer patients from one institution diagnosed in three consecutive years.

On the other hand, we acknowledge the limitations of our study. The relatively small sample size due to a single source of patient population can affect statistical power of the study. Our comparison did not account for the percentage replacement of LNs by CSCA and its association to the survival, which will serve as an objective in our future follow-up study. The information regarding local recurrence, metastatic disease, and chemoradiation therapy was not included in our study. However, previous studies have shown that LNR can be used in decision making when the aforementioned conditions are present [18]. In the cases without axillary LN dissection, that is, patients who underwent chemotherapy with the biopsy of only one positive sentinel LN (1/1), it was difficult to evaluate the LNR without sufficient information of the denominator. However, we still included these patients in our study owing to the comparison to other LN-related parameters (N stage and LN CSCA).

In conclusion, LNR categorizes breast cancer patients based on the ratio of the number of positive to total axillary LNs examined and is demonstrated to be the most powerful LN-related prognostic factor. It outperforms the other LN-related methods, such as the current LN staging system recognized by AJCC and the LN CSCA, in predicting breast cancer survival in our study. However, LN CSCA is still found to be a promising survival predicator in our pilot study. More extensive analysis in a large-scale study is needed to further define its role in breast cancer management.

## Figures and Tables

**Table 1:** Characteristics of breast cancer patients.

Characteristics	No. of patients *n* (%)
Total number	292
Mean age	59.0 ± 14.4
≤50	92
>50	200
N stage	
N0	129 (50%)
N1	81 (32%)
N2	26 (10%)
N3	20 (8%)
T stage	
T1	147 (51%)
T2	109 (38%)
T3	16 (6%)
T4	16 (5%)
Histologic grade	
G1	35 (12%)
G2	147 (51%)
G3	109 (37%)
Hormonal status	
ER+	220 (78%)
ER−	62 (22%)
ECE	
Present	80 (63%)
Absent	47 (37%)
LNR (node-positive, *n* = 127)	
<10%	43 (34%)
10–65%	60 (47%)
>65%	24 (19%)
LN CSCA (node-positive, *n* = 107)	
<50 mm^2^	47 (44%)
50–500 mm^2^	44 (41%)
>500 mm^2^	16 (15%)

**Table 2:** Prognostic factors and overall survival rate (univariate analysis by Kaplan-Meier).

	5-year OS% ± SE	*P* value
Age		
≤50	84 ± 4	0.6
>50	82 ± 2	
Histologic grade		
G1	90 ± 5	
G2	89 ± 3	0.001
G3	73 ± 4	
N stage		
N0	94 ± 2	
N1	86 ± 4	<0.0001
N2	53 ± 10	
N3	46 ± 12	
T stage		
T1	92 ± 2	
T2	84 ± 36	<0.0001
T3	43 ± 13	
T4	36 ± 12	
ER		
Yes	89 ± 2	<0.0001
No	62 ± 6	
ECE		
Yes	93 ± 20	<0.0001
No	63 ± 6	
LN CSCA (mm^2^)		
0 (N0)	94 ± 22	
<50	85 ± 54	<0.0001
50–500	65 ± 7	
>500	46 ± 13	
LNR		
0 (N0)	94 ± 22	
<0.1	90 ± 4	<0.0001
0.1–0.65	79 ± 5	
>0.65	28 ± 10	

**Table 3:** Multivariate analysis of the prognostic factors by Cox's regression model in node-positive patients (*n* = 107).

Variable	Hazard Ratio	95% CI	*P-*value
Age (continuous)	0.99	0.97–1.03	0.887
T1 versus (T2 + T3 + T4)	0.88	0.33–2.34	0.791
Tumor size (continuous)	1.12	0.99–1.26	0.071
Histologic grade (G1 + G2 versus G3)	**3.52**	**1.66**–**7.48**	**0.001**
ER expression (yes versus no)	**0.39**	**0.19**–**0.81**	**0.012**
ECE (yes versus no)	0.73	0.26–2.07	0.550
metLN (continuous)	0.93	0.01–1.018	0.052
LNR (continuous)	**25.02**	**5.37**–**116.59**	**0.001**
LN cancer area (continuous)	2.24	0.45–11.09	0.323

## References

[1] Jemal A, Siegel R, Xu J, Ward E (2010). Cancer statistics, 2010. *CA Cancer Journal for Clinicians*.

[2] Amersi F, Hansen NM (2006). The benefits and limitations of sentinel lymph node biopsy. *Current Treatment Options in Oncology*.

[3] Dunst J, Steil B, Furch S (2001). Prognostic significance of local recurrence in breast cancer after postmastectomy radiotherapy. *Strahlentherapie und Onkologie*.

[4] Eifel P, Axelson JA, Costa J (2001). National institutes of health consensus development conference statement: adjuvant therapy for breast cancer, November 1–3, 2000. *Journal of the National Cancer Institute*.

[5] Wilking N, Rutqvist LE, Carstensen J, Mattsson A, Skoog L (1992). Prognostic significance of axillary nodal status in primary breast cancer in relation to the number of resected nodes. *Acta Oncologica*.

[6] (1998). The Steering Committee on Clinical Practice Guidelines for the Care and Treatment of Breast Cancer. *Canadian Medical Association Journal*.

[7] Gipponi M, Bassetti C, Canavese G (2004). Sentinel lymph node as a new marker for therapeutic planning in breast cancer patients. *Journal of Surgical Oncology*.

[8] Kuru B, Camlibel M, Dinc S, Gulcelik MA, Alagol H (2003). Prognostic significance of axillary node and infraclavicular lymph node status after mastectomy. *European Journal of Surgical Oncology*.

[9] American Joint Committee on Cancer (2010). *Cancer Staging Manual*.

[10] Sivridis E, Giatromanolaki A, Galazios G, Koukourakis MI (2006). Node-related factors and survival in node-positive breast carcinomas. *Breast*.

[11] Gonzalez-Angulo AM, Morales-Vasquez F, Hortobagyi GN (2007). Overview of resistance to systemic therapy in patients with breast cancer. *Advances in Experimental Medicine and Biology*.

[12] Atahan IL, Ozyigit G, Yildiz F (2008). Percent positive axillary involvement predicts for the development of brain metastasis in high-risk patients with nonmetastatic breast cancer receiving post-mastectomy radiotherapy. *The Breast Journal*.

[13] Atahan IL, Yildiz F, Ozyigit G (2008). Percent positive axillary lymph node metastasis predicts survival in patients with non-metastatic breast cancer. *Acta Oncologica*.

[14] Tan YY, Fan YG, Lu Y (2005). Ratio of positive to total number of sentinel nodes predicts nonsentinel node status in breast cancer patients. *The Breast Journal*.

[15] Truong PT, Berthelet E, Lee J, Kader HA, Olivotto IA (2005). The prognostic significance of the percentage of positive/dissected axillary lymph nodes in breast cancer recurrence and survival in patients with one to three positive axillary lymph nodes. *Cancer*.

[16] Truong PT, Vinh-Hung V, Cserni G, Woodward WA, Tai P, Vlastos G (2008). The number of positive nodes and the ratio of positive to excised nodes are significant predictors of survival in women with micrometastatic node-positive breast cancer. *European Journal of Cancer*.

[17] van der Wal BCH, Butzelaar RMJM, van der Meij S, Boermeester MA (2002). Axillary lymph node ratio and total number of removed lymph nodes: predictors of survival in stage I and II breast cancer. *European Journal of Surgical Oncology*.

[18] Vinh-Hung V, Nguyen NP, Cserni G (2009). Prognostic value of nodal ratios in node-positive breast cancer: a compiled update. *Future Oncology*.

[19] Vinh-Hung V, Verkooijen HM, Fioretta G (2009). Lymph node ratio as an alternative to pN staging in node-positive breast cancer. *Journal of Clinical Oncology*.

[20] Vinh-Hung V, Verschraegen C, Promish DI (2004). Ratios of involved nodes in early breast cancer. *Breast Cancer Research*.

[21] Voordeckers M, Vinh-Hung V, van de Steene J, Lamote J, Storme G (2004). The lymph node ratio as prognostic factor in node-positive breast cancer. *Radiotherapy and Oncology*.

[22] Dabbs DJ, Fung M, Landsittel D, McManus K, Johnson R (2004). Sentinel lymph node micrometastasis as a predictor of axillary tumor burden. *The Breast Journal*.

[23] de Boer M, van Dijck JAAM, Bult P, Borm GF, Tjan-Heijnen VCG (2010). Breast cancer prognosis and occult lymph node metastases, isolated tumor cells, and micrometastases. *Journal of the National Cancer Institute*.

[24] Reed J, Rosman M, Verbanac KM, Mannie A, Cheng Z, Tafra L (2009). Prognostic implications of isolated tumor cells and micrometastases in sentinel nodes of patients with invasive breast cancer: 10-year analysis of patients enrolled in the prospective east carolina university/anne arundel medical center sentinel node multicenter study. *Journal of the American College of Surgeons*.

[25] van Deurzen CHM, de Boer M, Monninkhof EM (2008). Non-sentinel lymph node metastases associated with isolated breast cancer cells in the sentinel node. *Journal of the National Cancer Institute*.

[26] Yegiyants S, Romero LM, Haigh PI, DiFronzo LA (2010). Completion axillary lymph node dissection not required for regional control in patients with breast cancer who have micrometastases in a sentinel node. *Archives of Surgery*.

[27] Noguchi M (2008). Avoidance of axillary lymph node dissection in selected patients with node-positive breast cancer. *European Journal of Surgical Oncology*.

[28] Sanki A, Uren RF, Moncrieff M (2009). Targeted high-resolution ultrasound is not an effective substitute for sentinel lymph node biopsy in patients with primary cutaneous melanoma. *Journal of Clinical Oncology*.

[29] Scolyer RA, Li LXL, McCarthy SW (2004). Micromorphometric features of positive sentinel lymph nodes predict involvement of nonsentinel nodes in patients with melanoma. *American Journal of Clinical Pathology*.

[30] Goyal A, Douglas-Jones A, Newcombe RG, Mansel RE (2004). Predictors of non-sentinel lymph node metastasis in breast cancer patients. *European Journal of Cancer*.

[31] Komori T, Doki Y, Kabuto T (2003). Prognostic significance of the size of cancer nests in metastatic lymph nodes in human esophageal cancers. *Journal of Surgical Oncology*.

[32] Petrelli F, Borgonovo K, Barni S (2011). The emerging issue of ratio of metastatic to resected lymph nodes in gastrointestinal cancers: an overview of literature. *European Journal of Surgical Oncology*.

[33] Cil T, Hauspy J, Kahn H (2008). Factors affecting axillary lymph node retrieval and assessment in breast cancer patients. *Annals of Surgical Oncology*.

[34] Marrazzo A, Taormina P, Gebbiab V (2007). Is sentinel lymph node biopsy more accurate than axillary dissection for staging nodal involvement in breast cancer patients?. *Chirurgia italiana*.

